# A Direct Catalytic Ethanol Fuel Cell (DCEFC) Modified by LDHs, or by Catalase-LDHs, and Improvement in Its Kinetic Performance: Applications for Human Saliva and Disinfectant Products for COVID-19

**DOI:** 10.3390/bios13040441

**Published:** 2023-03-30

**Authors:** Mauro Tomassetti, Riccardo Pezzilli, Claudio Leonardi, Giuseppe Prestopino, Corrado Di Natale, Luigi Campanella, Pier Gianni Medaglia

**Affiliations:** 1 Department of Electronic Engineering, University of Rome “Tor Vergata”, Viale del Politecnico 1, 00133 Rome, Italy; 2 Department of Chemistry, University of Rome “La Sapienza”, P.le A. Moro 5, 00185 Rome, Italy; 3 Department of Industrial Engineering, University of Rome “Tor Vergata”, Viale del Politecnico 1, 00133 Rome, Italy

**Keywords:** modified direct catalytic ethanol fuel cell, efficiency improvement using LDHs, catalase enzyme crosslinking LDHs, application to check ethanol in saliva, disinfectant solutions

## Abstract

In this work, it has been experimentally proven that the kinetic performance of a common Direct Catalytic Ethanol Fuel Cell (DCEFC) can be increased by introducing nanostructured (Zn^II^,Al^III^(OH)_2_)^+^NO_3_^−^·H_2_O Layered Double Hydroxides (LDHs) into the anode compartment. Carrying out the measurements with the open-circuit voltage method and using a kinetic format, it has been shown that the introduction of LDHs in the anodic compartment implies a 1.3-fold increase in the calibration sensitivity of the method. This improvement becomes even greater in the presence of hydrogen peroxide in a solution. Furthermore, we show that the calibration sensitivity increased by 8-times, when the fuel cell is modified by the enzyme catalase, crosslinked on LDHs and in the presence of hydrogen peroxide. The fuel cell, thus modified (with or without enzyme), has been used for analytical applications on real samples, such as biological (human saliva) and hand disinfectant samples, commonly used for the prevention of COVID-19, obtaining very positive results from both analytical and kinetic points of view on ethanol detection. Moreover, if the increase in the calibration sensitivity is of great importance from the point of view of analytical applications, it must be remarked that the increase in the speed of the ethanol oxidation process in the fuel cell can also be extremely useful for the purposes of improving the energy performance of a DCEFC.

## 1. Introduction

It is known that, despite the global oxidation reaction, which takes place in the anodic section of a Direct Catalytic Ethanol (or methanol) Fuel Cell (DCEFC) exploiting metal catalysts, such as Pt, Ru, Pd, and so on, seeming relatively simple, it is actually characterized by a laborious series of reactions, which generally makes the complete oxidation of the alcoholic fuel by the cell quite slow [1,2].

For the cell used in this work, having catalysts of the Pt-Ru type, the anodic total reaction for ethanol and methanol can be, respectively, schematized, in the simplest and most concise way, as the following:

(1) $$C_{2}H_{5}\mathrm{OH}+3H_{2}O\;\overset{\;\;\;\;\;\;\;\;\;\;\;\;\;\;\;\;\;\;\;\;\;\;\;\;\;\;\;}{\to }\;12H^{+}+12e^{-}+2{\mathrm{CO}}_{2}$$

and:

(2) $${\mathrm{CH}}_{3}\mathrm{OH}+H_{2}O\;\overset{\;\;\;\;\;\;\;\;\;\;\;\;\;\;\;\;\;\;\;\;\;\;\;\;\;\;\;}{\to }\;6H^{+}+6e^{-}+{\mathrm{CO}}_{2}$$

However, as mentioned above, this is accomplished through several processes. The first steps transform ethanol (or methanol) into oxidized derivatives are generally the slowest ones [1,2,3]. In the literature, it is already reported that some types of LDH (Layered Double Hydroxide) compounds can catalyze the oxidation of ethanol in an alkaline environment [4], according to the following reaction:

$${\mathrm{CH}}_{3}{\mathrm{CH}}_{2}\mathrm{OH}+3{\mathrm{OH}}^{-}\;\overset{\;\;\;\;\;\;\;\;\;\;\;\;\;\;\;\;\;\;\;\;\;\;\;\;\;\;\;}{\to }\;{\mathrm{CH}}_{3}{\mathrm{CO}}_{\left(\mathrm{ads}\right)}+3H_{2}O+3e^{-}$$

On the other hand, it is widely reported that in the presence of Pt alone, during the catalytic oxidation reaction of methanol, even in a neutral environment, intermediate oxidized species are formed, such as CH_2_O [5,6], according to a reaction that is not very fast [7]:

$${\mathrm{Pt-CH}}_{2}\mathrm{OH}\;\overset{\;\;\;\;\;\;\;\;\;\;\;\;\;\;\;\;\;\;\;\;\;\;\;\;\;\;\;}{\to }\;\mathrm{Pt}(s)+\mathrm{HCHO}+H^{+}+e^{-}$$

While, in the case of ethanol, it can probably be written analogously as:

$${\mathrm{Pt-CH}}_{3}{\mathrm{CH}}_{2}\mathrm{OH}\;\overset{\;\;\;\;\;\;\;\;\;\;\;\;\;\;\;\;\;\;\;\;\;\;\;\;\;\;\;}{\to }\;\mathrm{Pt}(s)+{\mathrm{CH}}_{3}\mathrm{CHO}+2H^{+}+2e^{-}$$

or even [8]:

$${\mathrm{CH}}_{3}{\mathrm{CH}}_{2}\mathrm{OH}\;\overset{\;\;\;\;\;\;\;\;\;\;\;\;\;\;\;\;\;\;\;\;\;\;\;\;\;\;\;}{\to }\;{\mathrm{CH}}_{3}{\mathrm{CHO}}_{\mathrm{ads}}\;\overset{\;\;\;\;\;\;\;\;\;\;\;\;\;\;\;\;\;\;\;\;\;\;\;\;\;\;\;}{\to }\;{\mathrm{CH}}_{3}\mathrm{CHO}+2H^{+}+2e^{-}$$

In recent years, a certain number of studies, based on LDHs of the type (Zn^II^,Al^III^(OH)_2_)^+^NO_3_^−^·H_2_O, were conducted by our research group, obtaining good results, especially from an analytical point of view [9,10,11,12]. Furthermore, encouraging results have been obtained, both analytical, e.g., on alcoholic drinks, beverages [13,14,15], drugs, or pharmaceutical formulations [16,17,18,19,20,21], and also for energy purposes with the use of glucose and carbohydrates [15,22] by means of a simple and inexpensive DCEFC. We, therefore, came up with the idea of trying to accelerate the catalytic reaction of our fuel cell by inserting our previously used LDHs, housed inside a small dialysis tube, into the anodic section of the fuel cell (already containing metals, such as Pt and Ru) [9,10,11]), as already carried out by our team but using an enzyme instead of LDHs [14]. In other words, the basic idea is to realize an extremely simple and inexpensive device, starting from the same fuel cell already used in previous works, without essential modifications [13,14,15,16,17,18,19,20,21,22]. However, in this case, we operated in open-circuit mode, rather than in a potentiostatic way, since this mode has two undoubted advantages: firstly, it proved to be very reproducible and, secondly, the method does not necessarily require one to determine the Optimized Applied Potential (OAP) [13,23,24]. Indeed, the OAP determination may cause inaccuracy and poor reproducibility of the measurement, as it can undergo variations upon repeated measurements, especially on real samples. Thus, operating in open-circuit mode and recording the experimental trend of the “charge curves” of the cell potential, we realized that, using a kinetic format, it is possible to prove the proposed hypothesis, i.e., the possibility to accelerate the anodic catalytic process of our fuel cell by inserting a weighted amount of our LDHs, within a small dialysis tube, into its anodic section, already containing Pt and Ru metals, and using ethanol as fuel. Therefore, the experiments performed, using this open-circuit kinetic format and fuel cell containing LDHs, we obtained positive results, which became even better in the presence of hydrogen peroxide in fuel solution. Subsequently, a further increase in the kinetic performance of the fuel cell was obtained by immobilizing the catalase enzyme on our LDHs through adsorption and subsequent crosslinking processes, as in our previous works [9,10], obtaining even better results.

## 2. Materials and Methods

### 2.1. Apparatus

The commercial fuel cell used in this research was made in Plexiglas^®^; the electrodes were made in Pt-Ru black catalyst and a Nafion™ polymeric exchange membrane (PEM). As a typology, a (DCEFC) Direct Catalytic Ethanol (or Methanol) Fuel Cell could be defined, marketed as H-TEC F111 by Fuel Cell Store (College Station, TX, USA). To carry out the measurements, the Fuel Cell was connected to a potentiostat mod. Em-Stat, provided by Palmsens (Houten, The Netherlands), and to a PC, by means of PSTrace Software version 4.6 data interface.

### 2.2. Measurement Format

The fuel cell operates in open-circuit and in batch mode, recording the increase in the voltage charge curve. For each measurement, after a hydroalcoholic solution (about 2 mL) with a fixed concentration of ethanol was introduced into the fuel cell, the open-circuit voltage value was immediately and continuously recorded, until a constant-maximum potential was reached. At this point, the slope value of the tangent line at the point of maximum slope to the obtained curve was found.

**Figure 1:**
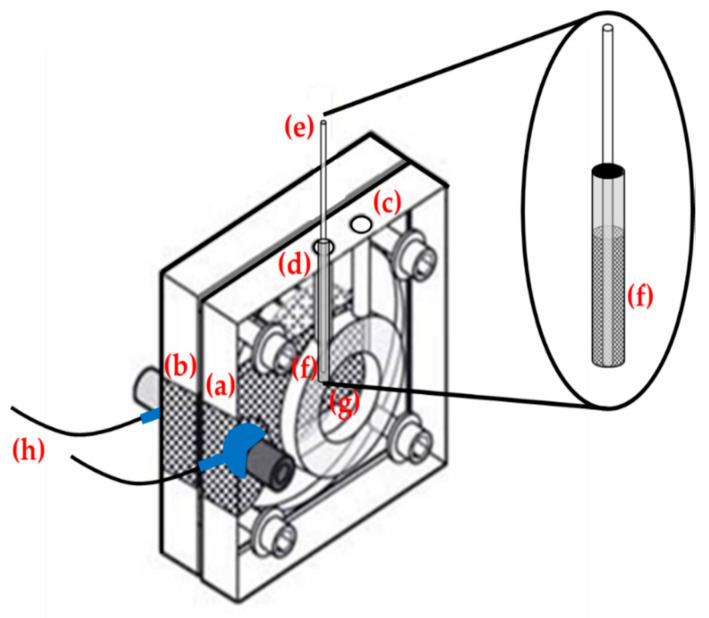
Schematic representation of the fuel cell used, where a small cylindrical dialysis membrane containing LDHs was inserted in the anodic section: (**a**) Anodic section of fuel cell. (**b**) Cathodic section of fuel cell. (**c**) Filling hole of the fuel cell with hydroalcoholic solution. Diameter 3.5 mm. (**d**) Hole containing the small cylindrical dialysis membrane (diameter of about 3.4 mm) containing LDHs, immersed 3 cm in the hydroalcoholic solution. (**e**) Holder of the cylindrical dialysis membrane with a diameter of approx. 2 mm. (**f**) Gap containing 63 mg of LDHs. (**g**) Cavity containing the two circular Pt-Ru electrodes, separated by a graphitized Nafion membrane. (**h**) To Em-Stat potentiostat.

Each measurement, thus, was carried out first by recording the potential curve, operating with the cell as such, filled with hydroalcoholic solution. Subsequently, a second curve was recorded in identical conditions, but introducing about 63 mg of LDHs, within a small cylindrical dialysis membrane (i.e., the maximum amount of LDH that could be contained in the cylindrical dialysis membrane) into the anodic section of the cell, for the entire duration of the measurement, as shown in Figure 1.

The same apparatus (modified fuel cell) shown in Figure 1 was also used with the measurements in the presence of the catalase enzyme. The immobilization of catalase in LDHs of the (Zn^II^,Al^III^(OH)_2_)^+^NO_3_^−^·H_2_O type was carried out by adding three different amounts, i.e., 40 mg, 60 mg, and 90 mg of catalase (powder) to 63 mg of LDHs. The two components were carefully homogenized through the addition of one–two micro drops of 0.1 mol L^−1^ pH 7 phosphate buffer solution.

The small quantity of paste, thus obtained, was placed in a glass crystallizer with an emery lid. A small open glass capsule (1.5 cm in diameter), containing 30 mg of glutaraldehyde (25%), was also placed inside the crystallizer. The crystallizer was then closed by means of its lid and the glutaraldehyde drops were allowed to evaporate, so that the crosslinking reaction between the nanostructure of LDH and the catalase absorbed on the latter took place by glutaraldehyde. After placing the closed crystallizer in the refrigerator for a few hours, the obtained paste granules were quantitatively placed inside the small cylindrical dialysis membrane and kept stretched by a small rigid support placed inside. Finally, the small cylindrical membrane was sealed and inserted into one of the two cylindrical holes of the fuel cell.

A first set of measurements was carried out with only LDHs in the dialysis membrane and the fuel cell filled with 2.50% ethanol hydroalcoholic solution; in a second set of measurements, the LDH-catalase compound was employed and a fixed concentration of H_2_O_2_ (12 mmol L^−1^) was added in the hydroalcoholic solution. In both cases, the increase in the potential, generated between the respective anodic and cathodic ends of the fuel cell, was recorded.

### 2.3. Measurements in Real Samples by Fuel Cell

For the determination of ethanol in saliva: two different samples of 5.0 g of human saliva were centrifuged for 10 min. Then, 3.2 g of each was brought to 5.0 g by adding distilled and deionized water; 25 µL of H_2_O_2_ at 10% by volume and two different amounts of ethanol, 0.004 g or 0.008 g, respectively, were rapidly added to both solutions, thus obtaining two biological samples at different concentrations.

Approximately 2.0 mL of each solution was transferred into the anodic compartment of the modified fuel cell with crosslinked catalase on LDHs. During the addition, we started to record the potential difference between anode and cathode of the fuel cell, operating in open circuit.

The percentage of ethanol in saliva samples was recovered using a constructed calibration curve of ethanol (CAS, 99.9% by volume) solution, in a range 0.015–0.29%, and a fixed concentration of H_2_O_2_ (12 mmol L^−1^).

For the determination of ethanol in anti-COVID-19 disinfectant samples, the three different commercial solutions were first diluted 1:20 with distilled and deionized water and homogenized; then, 25 µL of H_2_O_2_ 10% by volume was added to each solution. Approximately 2.0 mL of each solution was transferred into the anodic compartment of the modified fuel cell with only LDHs. During the addition, the potential difference between anode and cathode of the fuel cell, operating in open circuit, was recorded.

The percentage of ethanol in anti-COVID-19 disinfectant samples was obtained using a specifically constructed calibration curve with denatured alcohol at 90% by volume, in a range 1.75–9.00%, and a fixed concentration of H_2_O_2_ (12 mmol L^−1^).

Lastly, all the numerical values reported in this work are always the average of at least three repeated experimental determinations.

### 2.4. Reagents, Materials, and Real Samples

The dialysis membrane (D-9777, Sigma-Aldrich, Steinheim, Germany) and catalase from bovine liver (EC 1.11.1.6) were provided by Sigma-Aldrich (Steinheim, Germany); hydrogen peroxide RPE at 30% *v*/*v*, by Carlo Erba (Milan, Italy) and 3% *v*/*v*, by AIESI (Naples, Italy) Ethanol (CAS: 64-17-5), 99.9% purity, was supplied by Sigma Aldrich (Milan, Italy), while denatured ethanol, 90% by volume, was purchased in local pharmacies (Rome, Italy). Zinc nitrate hexahydrate (Zn (NO_3_)_2_·6H_2_O) and aluminum nitrate nonahydrate (Al (NO_3_)_3_·9H_2_O) for LDH preparation were both from Sigma-Aldrich (Steinheim, Germany).

The used hydroalcoholic ethanol (or denatured ethanol) working solutions were obtained by properly diluting, with distilled and deionized water (final conductivity 0.01–0.02 µS), known volumes of ethanol.

The real samples analyzed were: (a) three sealed bottles, containing the commercial anti-COVID-19 disinfectant solutions most used in Italy, produced by the Italian pharmaceutical industry and purchased in public drug stores, in which the concentrations of denatured alcohol declared by the manufacturers were, respectively, equal to: 66%, 70%, and 74%, as well as minimal unquantified traces of other excipients, such as propylene glycol, glycerin, imidazolidinyl urea, and natural flavors; (b) two samples, approximately 10 mL, of human saliva, donated immediately before the measurements by two of the authors of this article (aged 80 and 30, respectively, both male, and both recognized by the health authority as healthy).

### 2.5. LDH Preparation and Characterization

To obtain the (Zn^II^,Al^III^(OH)_2_)^+^NO_3_^−^·H_2_O LDHs type, hereinafter also written several times as (Zn–Al–NO_3_), in this work, we used the co-precipitation method for the synthesis of LDHs, described in more detail in our previous works [11,12]. Briefly, to carry out the synthesis of LDH of the type (Zn-Al-NO_3_): 50 mmol L^−1^ of aluminum nitrate and 150 mmol L^−1^ of zinc nitrate were dissolved in 200 mL of distilled and deionized water. The pH of this solution was then adjusted to pH = 10 by means of NaOH; then, it was placed in well-sealed container and kept for 12 h in an oven, maintained at a temperature of 90 °C. The precipitation of LDH occurred during this time period. The solution containing the precipitate was cooled then centrifuged at 3000 revolutions per minute for about 12 min. At the end, the precipitate was separated from the solution and washed repeatedly, first with ethanol, then with distilled and deionized water. Finally, the solid, thus obtained, was dried at a temperature of 45 °C and immediately used or stored in a closed container at room temperature.

The characterization of the product obtained was carried out using an X-ray Diffraction spectrometer (XRD) and other instrumental methods: Fourier-Transform Infrared Spectroscopy (FT-IR), Thermo-Gravimetric analysis (TGA), Differential Thermal Analysis (DTA), and Scanning Electron Microscopy (SEM), described and reported in previous works [10,11,25,26].

## 3. Results and Discussion

### 3.1. Modification of Fuel Cell to Increase Its Performance

As already mentioned, to prove the assumptions of this research, several voltage charge curves of the fuel cell were constructed and recorded, operating in open-circuit mode, using different concentrations of hydroalcoholic solutions of ethanol, ranging between 1.25% and 15.0%. Each experiment was firstly carried out with the anodic section of the fuel cell containing only the hydroalcoholic solution and subsequently repeating experiments with 63 mg of (Zn–Al–NO_3_), within a small cylindrical dialysis membrane inserted in the fuel cell, filled with the same hydroalcoholic solution.

**Figure 2:**
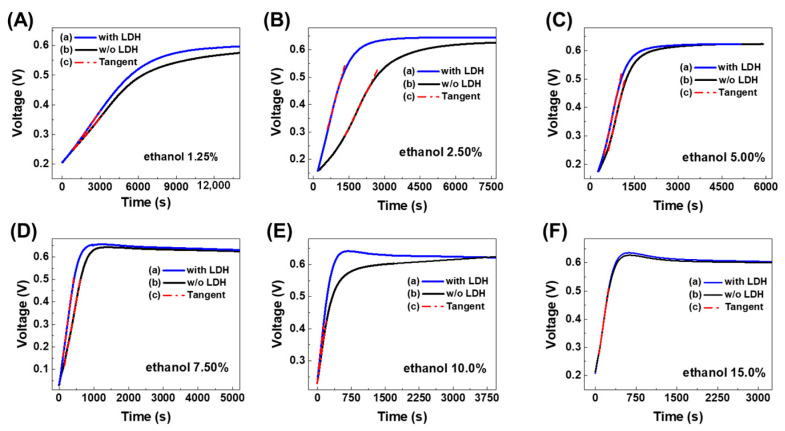
Open-circuit fuel cell voltage charge curves as voltage vs. time, performed by operating with different percentages of hydroalcoholic solution: (**A**) 1.25%, (**B**) 2.50%, (**C**) 5.00%, (**D**) 7.50%, (**E**) 10.0%, and (**F**) 15.0%. Each figure shows the tangent line (c) at the point of maximum slope to the obtained curves: (a) in the presence of LDHs (63 mg), (b) without LDHs.

The voltage charge curves, with and without LDHs, and the tangent line at the point of maximum slope to the obtained curves, for each concentration of hydroalcoholic solution, are represented in Figure 2.

**Figure 3:**
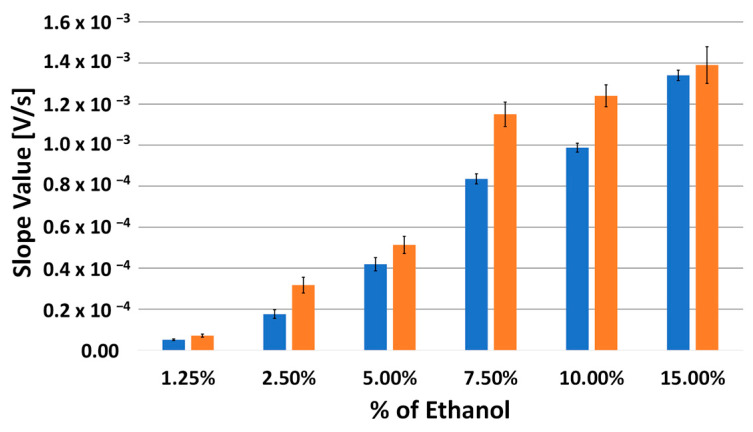
Comparison by bar chart diagram of the slope values of the tangent line at the point of maximum slope to the obtained curves, without (blue bars) and with (orange bars) LDHs (63 mg), at the different percentages of hydroalcoholic solution used.

The slope values, with and without LDHs, were calculated and are compared in Table 1 and as a bar chart diagram in Figure 3, for each of the different percentages of hydroalcoholic solution used. This represents a characteristic index of the speed of the charging process of the system under examination.

The slope values, reported both in Table 1 and in Figure 3, were calculated in a range of ethanol in hydroalcoholic solution, between a minimum value of 1.20% and a maximum value of 15.0%. This interval is not small, considering the fact that the maximum concentration recommended by the manufacturer should not exceed higher alcohol concentrations than about 3%, even though, in previous works, it was possible to ascertain that our fuel cell could operate even at significantly higher ethanol concentrations.

**Figure 4:**
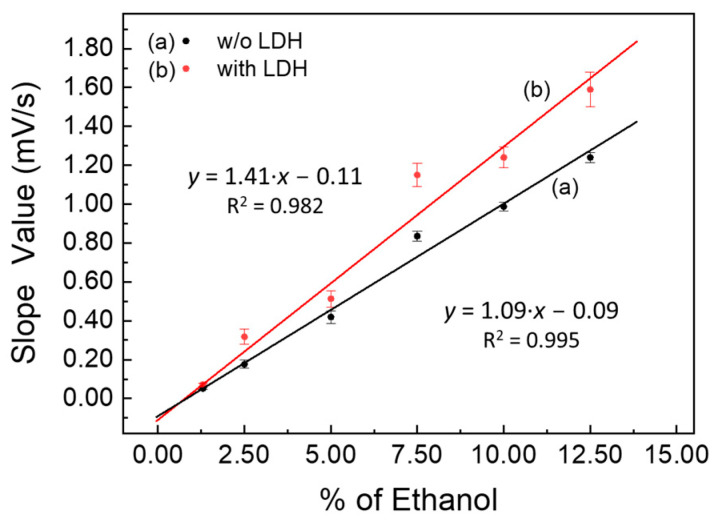
Calibration curves by «kinetic methods» (slope values vs. increasing percentage of ethanol), both in the absence (**a**) and in the presence of LDHs (63 mg) (**b**).

Two calibration curves, displayed in Figure 4, recorded both in the absence and presence of LDHs using the slope values reported in Table 1, were obtained.

A constant-maximum value of potential difference generated by the fuel cell, equal to 612 mV, is reached with a speed of charging proportional to the percentage of ethanol contained in the hydroalcoholic solution, as shown in Figure 2.

Furthermore, the most interesting thing is that, comparing the slope values reported in Table 1, in Figure 3, and in Figure 4, they are higher in the presence of LDHs for each hydroalcoholic solution tested. This means that the speed of charging of the fuel cell was essentially greater in the presence than in the absence of LDHs. In conclusion, the presence of LDHs exerts a catalytic effect on the oxidative process of ethanol, which was added to the catalytic one due to the Pt-Ru of the electrodes in the Fuel Cell. Indeed, the catalytic properties of LDH nanostructures are well documented in the literature, in a plethora of chemical processes (i.e., oxidation of organic substances, air pollution elimination, oxidation of carbohydrates, dehydrogenation reactions [27], but above all catalytic oxidation of alcohols to low temperatures [28]). It is also observed that the increase in the speed of the catalytic process, due to the presence of LDHs, is more evident in more diluted hydroalcoholic solutions. This is naturally due to the fact that, at high percentages of ethanol, the overall catalytic oxidation rate increases significantly, and it becomes more difficult to separate the catalytic contributions provided by the presence of LDHs from those obtained with the Pt-Ru electrodes.

**Figure 5:**
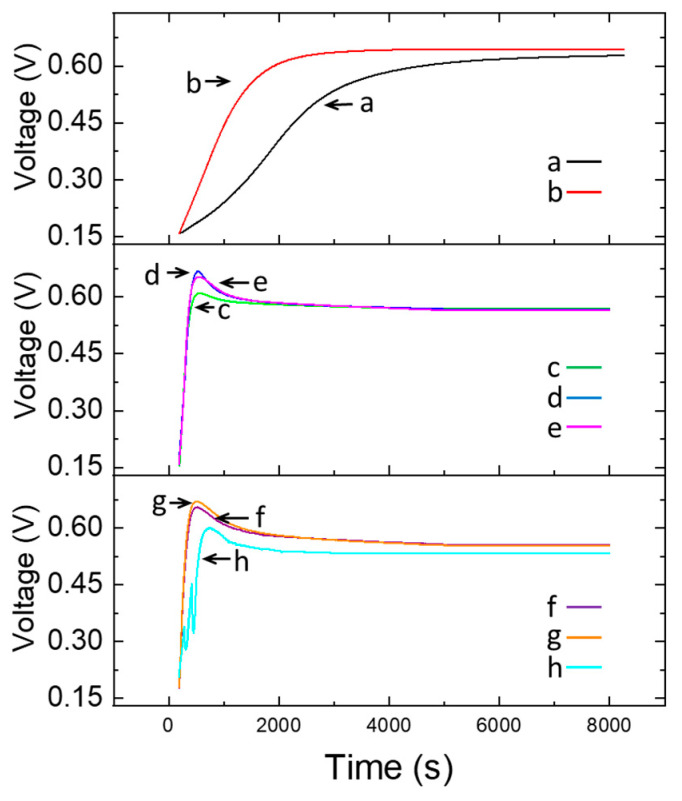
Differences in the response of the fuel cell, fed with 2.50% ethanol hydroalcoholic solution: (**a**) as such, (**b**) modified with LDHs, i.e., ZnAl-NO_3_ (63 mg), inserted into the anodic section, (**c**) modified with LDH-catalase mixture (63 mg: 40 mg) inserted into the anodic section, (**d**) modified with LDH-catalase mixture (63 mg: 63 mg) inserted into the anodic section, and (**e**) modified with LDH-catalase mixture (63 mg: 92 mg) inserted into the anodic section. Furthermore, (**f**–**h**) measurements were carried out with fuel cell modified with LDH-catalase mixture (63 mg: 92 mg) but in the presence of different concentrations of hydrogen peroxide in solution, i.e., 4, 12, and 40 mmol L^−1^, respectively.

Once the positive effect of LDHs of the Zn-Al-NO_3_ type on the catalytic oxidation of ethanol had been ascertained, we wanted to verify experimentally whether the enzyme catalase, which, as is known, can, by itself, catalyze the oxidation of ethanol [29], once immobilized on LDHs by adsorption and crosslinking by means of glutaraldehyde vapors, can further increase the rate of catalytic oxidation of ethanol. For this purpose, experiments were carried out by placing within the dialysis membrane, inserted in the anodic section of the fuel cell, the LDH-catalase mixture, according to the weight ratios (1:0.7 *w*/*w*), or (1:1 *w*/*w*), or (1:1.5 *w*/*w*), as shown in Figure 5, where the open-circuit fuel cell loading curves, as voltage vs. time, were recorded at a fixed concentration of 2.50% ethanol in hydroalcoholic solution.

Finally, the last three measurements reported in Figure 5 were obtained in the same operating conditions but also in the presence of different concentrations of hydrogen peroxide in solution, i.e., 4, 12, and 40 mmol L^−1^, respectively.

**Figure 6:**
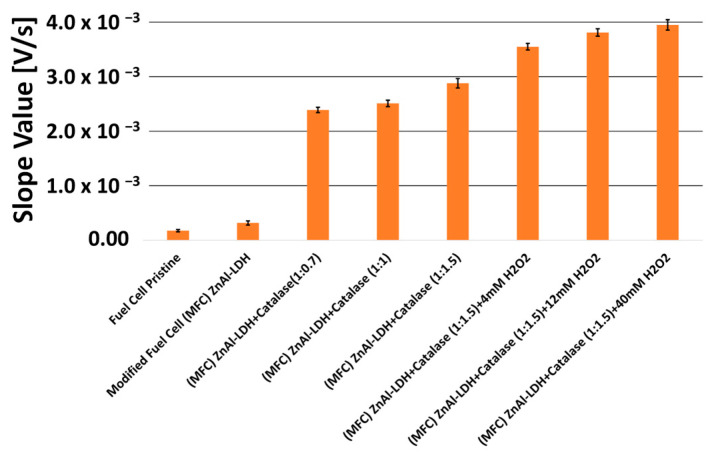
Comparison via bar chart diagram of the slope values of the tangent line at the point of maximum slope to the obtained voltage–charge curves, without or with LDHs, or with the addition of catalase enzyme at different weight ratio (1:0.7; 1:1; 1:1.5), or in the presence of different concentrations of hydrogen peroxide in solution, i.e., 4, 12, and 40 mmol L^−1^.

In Figure 6, a comparison can be observed in the form of a bar chart diagram, showing the slope values of the tangent line at the point of maximum slope to the obtained voltage–charge curves reported in Figure 5.

It is possible to observe how the presence of catalase causes a considerable increase in the rate of catalytic oxidation of ethanol, both for LDHs modified and, obviously, for a pristine Fuel Cell. Moreover, the increase in the catalytic effect is proportional to the ratio by weight (catalase/LDHs), although the response variations due to the different weight ratio are almost negligible when compared to the huge increase due to only the presence of the catalase enzyme itself. In practice, it seems that the strong increase in the catalytic velocity is due, above all, to the presence of the enzyme, rather than the relative increase in its quantity, crosslinked on the fixed quantity of LDHs.

The last three results reported in Figure 6, obtained in the presence of the catalase enzyme and hydrogen peroxide in solution, can probably be interpreted starting from this idea: the catalase enzyme exerts its catalytic capacity on the oxidation of ethanol, essentially when H_2_O_2_ is also present in solution, according to reactions:

(3) $$\mathrm{Cat}+H_{2}O_{2}\;\overset{\;\;\;\;\;\;\;\;\;\;\;\;\;\;\;\;\;\;\;\;\;\;\;\;\;\;\;\;\;\;\;\;\;\;\;\;\;}{↔}\;{\mathrm{Cat-H}}_{2}O_{2}$$

(4) $${\mathrm{Cat-H}}_{2}O_{2}+{\mathrm{CH}}_{3}{\mathrm{CH}}_{2}\mathrm{OH}\;\overset{\;\;\;\;\;\;\;\;\;\;\;\;\;\;\;\;\;\;\;\;\;\;\;\;}{\to }\;\mathrm{Cat}+2H_{2}O+{\mathrm{CH}}_{3}\mathrm{CHO}$$

in accordance with the literature [30,31]. However, in the presence of the catalase enzyme alone (i.e., without hydrogen peroxide), the catalytic effect should almost not be detected; as can be seen from what is reported in Figure 5 and Figure 6, a non-negligible catalytic effect on the oxidation of ethanol is clearly produced, as previously observed by some of the authors of this article [16].

We think that the explanation of this result might be found, most likely, in the formation of a certain amount of hydrogen peroxide in the aqueous solution, thanks to the presence of LDHs and, more specifically, thanks to the presence of metal ions contained in these layered crystals, according to reactions of the following type:

O_2_ + 2(H^+^ + e^−^) → H_2_O_2_

as reported in the literature [32,33].

Even the presence of carbonaceous material present on the sides of the Nafion membrane of the fuel cell seems able to facilitate the formation of H_2_O_2_, as well as the pH value itself [34], in which our fuel cell operates, i.e., around neutrality, where the CO_2_ in solution is essentially present in the form of bicarbonate.

On the other hand, the formation of some superoxides and hydroxyl radicals has been effectively found, for example, in the presence of some types of LDHs, even if ternary [35].

This could also explain the fact that, as observed in the experiments we carried out, the increase in the amount of catalase enzyme crosslinked inside the dialysis membrane seems to have a rather modest effect. It can probably be assumed that the concentration of the H_2_O_2_ formed is the limiting factor, even when it comes into contact with a greater and greater growing quantity of enzyme.

The catalytic effect of the catalase enzyme in ethanol oxidation, in the presence of hydrogen peroxide, can also be confirmed by the results reported by the last three bars in Figure 6, where additions of H_2_O_2_ in solution further increase the speed of the ethanol oxidation reaction and also the kinetic response of the fuel cell.

Moreover, Figure 5 and Figure 6 show that the kinetic response of the fuel cell naturally increases with the quantity of hydrogen peroxide added in solution, as expected. However, it is not advisable to increase the concentration of H_2_O_2_ in solution above a certain value, at least in the operating conditions used by us, in order to avoid the formation of gas bubbles (probably O_2_, according to reaction (4) shown above), leading to the formation of a strait of gas around the dialysis membrane; as a consequence, the solution contained in the fuel cell could be unable to freely penetrate inside the small cylindrical dialysis membrane, thus limiting a further increase in the speed of the catalytic reaction.

This aspect is clear also when we analyze the curve (h) in Figure 5, where the voltage response of the system seems to collapse rapidly when the gas bubbles give rise to a large bubble of O_2_ around the dialysis membrane and immediately increases again, as soon as this large O_2_ concentration around the membrane finally detaches from the membrane itself.

Therefore, at least in our experimental conditions, the most convenient concentration of hydrogen peroxide in solution to obtain a sufficiently high reaction rate, without significant disturbances from the gas bubbles produced by the catalytic reaction itself, should be about 12 mmol L^−1^.

**Figure 7:**
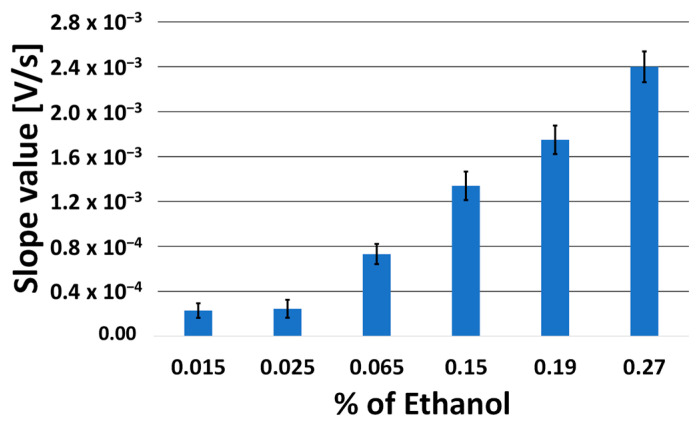
Bar chart diagram of the slope values of the tangent line at the point of maximum slope to the obtained voltage–charge curves, using catalase crosslinked with LDHs (1:1.5 by weight) and H_2_O_2_ (12 mmol L^−1^) in solution at different increasing ethanol percentages in the hydroalcoholic solutions tested.

Figure 7 reports the slope values, expressed as a bar chart diagram, of the tangent line at the point of maximum slope to the voltage–charge curves obtained using fixed 1:1.5 LDHs/enzyme ratio and keeping the concentration of H_2_O_2_ constant in solution, equal to 12 mmol L^−1^, at different percentages of ethanol in hydroalcoholic solution, ranging between 0.015% and about 0.29%. The considered low concentration interval is, in fact, the most useful for carrying out alcoholometric determinations in human saliva, such as those described in the following paragraph.

**Figure 8:**
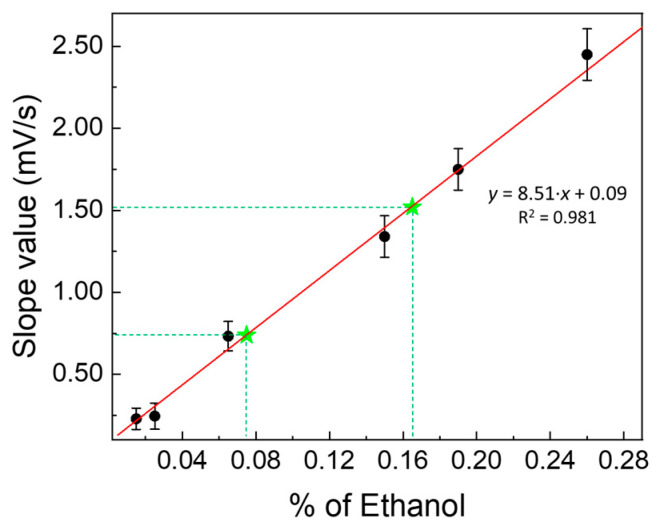
Calibration curves used for ethanol determination in human saliva samples. Calibration curve obtained by «kinetic methods» (slope values vs. increasing percentage of ethanol), using the enzymatic fuel cell modified by catalase, crosslinked with LDHs (1:1.5 by weight) and H_2_O_2_ (12 mmol L^−1^) in solution, in the hydroalcoholic solutions tested. Green stars represent the percentage of ethanol found in biological samples (saliva).

These operating conditions allowed to construct a calibration curve, shown in Figure 8, used precisely for this purpose (i.e., measurements at low concentrations, such as those present in saliva); in fact, it can be observed that the slope value of this enzymatic calibration curve is approximately 6-times greater than the slope value of the calibration curve obtained in the presence of only LDHs (Figure 4) in the dialysis membrane, inserted in the fuel cell (i.e., without enzyme or H_2_O_2_) and by almost 8-times greater than the slope of the calibration curve obtained with the fuel cell, i.e., without any modifications made in its anode compartment (see Figure 4).

### 3.2. Applications on Real Samples Using the Suitably Modified Fuel Cell

A series of applications, in previous research, was carried out by employing a non-modified fuel cell on real matrices, consisting of alcoholic beverages, such as different types of wines, beers, and spirits, or drugs (hydroalcoholic extracts, etc.), containing significant percentages of ethanol [14,15,16,17,18,19,20,21,22]. In the present work, the development of modified fuel cells, especially enzymatic, based on catalase immobilization on LDHs through adsorption and crosslinking mechanisms, ensured not only a considerable operational duration greater than at least one month but also a considerable increase in the calibration sensitivity by almost 8-times compared to the simple unmodified fuel cell, as mentioned in the previous paragraph. Above all, it allowed us to propose a method to determine the percentage of ethanol in human saliva.

In fact, it is known that with normal breathalyzer tests, based on photometric measurements [36,37], determinations are made on saliva samples containing concentrations of ethanol ranging between approximately 4 × 10^−3^ and 5 × 10^−2^ mol L^−1^ (corresponding to ethanol percentages between 23.3 × 10^−3^% and 29.1 × 10^−2^%). Since the calibration curve in Figure 8 is sufficiently linear between approximately 15 × 10^−3^% and 29.2 × 10^−2^%, it can certainly be used for suitable determinations of ethanol content in saliva.

We, therefore, carried out measurements of this type, using the format described in the Section 2.3, adding proper percentages of ethanol to human saliva, i.e., operating at ethanol percentages belonging to the range of interest (see Figure 8) found in the alcoholometric determinations in saliva, reported in the literature [36,37].

Table 2 reports the determination of ethanol concentration in diluted real samples, where it can be observed that the difference between the percentages of nominal ethanol and those measured experimentally never exceeds around 4.3%; this is acceptable accuracy for such complex and diluted biological samples.

Furthermore, we made other determinations regarding a very current requirement, i.e., the possibility to check the content of ethanol in alcohol-based disinfectant solutions, placed on the market to prevent COVID-19 infection.

**Figure 9:**
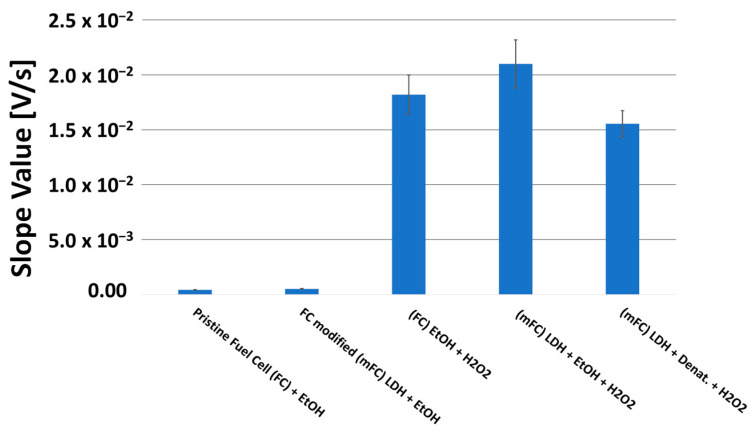
Comparison by bar chart diagram of the slope values of the tangent line at the point of maximum slope to the voltage–charge curves, obtained by fuel cell, fed with ethanol solution: pristine, modified with LDHs, pristine with H_2_O_2_ in solution (12 mmol L^−1^), modified with LDHs and H_2_O_2_ in solution (12 mmol L^−1^), and modified with LDHs and H_2_O_2_ in solution (12 mmol L^−1^), but fed with denatured ethanol (90% by volume) instead of 99.9% by volume of ethanol.

In this case, due to the high ethanol percentage in tested solutions, it was not necessary to use the enzymatic fuel cell but was sufficient to use a modified one with only LDHs. However, since it was observed, in some side tests, that adding H_2_O_2_ in solution to an LDH-modified fuel cell is able to increase the sensitivity of the measurement, in a non-negligible way, i.e., to enhance the oxidation kinetics of ethanol, we made our measurements by using hydrogen peroxide (see the bar chart diagram shown in Figure 9).

On the other hand, it is well documented in the literature that H_2_O_2_ is able to oxidize ethanol, especially in the presence of metal catalysts, such as Pt, or Au, or ions of Fe, Co, Cu [38], or complexes of ruthenium ion [39]. Therefore, it was foreseen that the catalytic effect of the fuel cell due to the platinum and ruthenium present in the anode benefited from the presence of hydrogen peroxide in solution. Thus, we decided to carry out the ethanol determinations in the disinfectant samples using this last configuration of the modified fuel cell, i.e., containing both LDHs and hydrogen peroxide in solution.

However, for this purpose, it is not possible to use a calibration curve built using ethanol (CAS, 99.9% purity, supplied by Sigma Aldrich) as fuel; rather, it is necessary to use denatured ethanol at 90.0% by volume, since commercial disinfectant solutions for COVID-19 contain considerable percentages of ethanol, but of the denatured type, with additives, such as b-denatonium benzoate, thiophene, methyl ethyl ketone, and, in some cases, a red dye (C.I. Red 24). These additive components can interfere with the catalysis of ruthenium ion (especially denatonium and red dye) [40] and reduce the response of the fuel cell to ethanol, albeit not dramatically [20]. The interference and reduction in the effects are confirmed by comparing the last two bars representing the slope values of the voltage–charge curves provided by the fuel cell fed with ethanol and denatured ethanol (see Figure 9).

**Figure 10:**
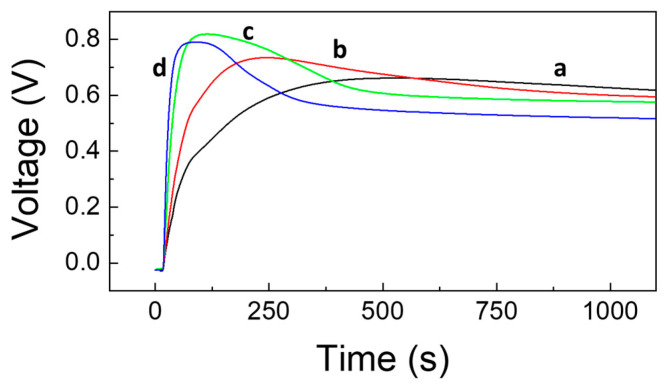
Voltage–charge curves as voltage vs. time, performed by operating with different percentages of denatured ethanol in the presence of LDHs and hydrogen peroxide in solution. Curves obtained using: (**a**) 1.92%, (**b**) 3.60%, (**c**) 7.00%, and (**d**) 9.00% in denatured ethanol.

**Figure 11:**
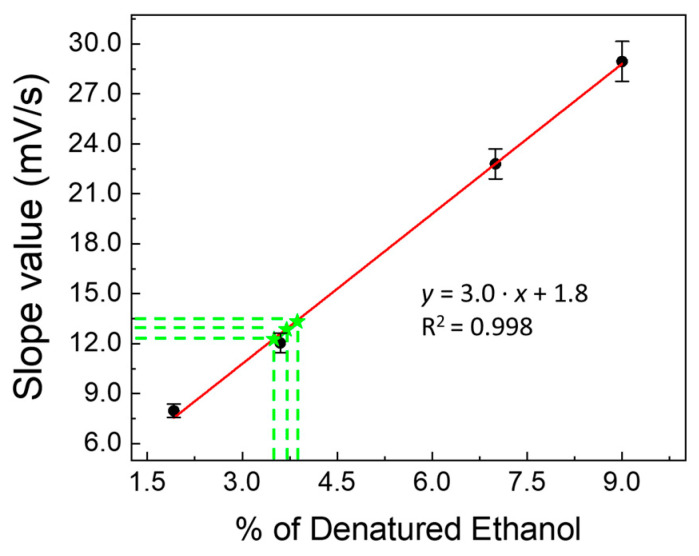
Calibration curves used for ethanol determination in samples of disinfectant solution for COVID-19. Calibration curve obtained by «kinetic methods» (slope values vs. increasing percentage of denatured ethanol), using the fuel cell modified by LDHs (63 mg) and H_2_O_2_ (12 mmol L^−1^) in solution in denatured ethanol solutions tested. Green stars represent found percentage values of ethanol in disinfectant samples.

Consequently, a new calibration curve was constructed, using the LDH-modified fuel cell in the presence of H_2_O_2_ (12 mmol L^−1^) and using increasing percentages of denatured ethanol instead of 99.9% ethanol as fuel. In Figure 10, we report the recorded voltage–charge curves of the fuel cell, as voltage vs. time, performed by operating with different percentages of denatured ethanol, that is: (a) 1.92%, (b) 3.60%, (c) 7.00%, and (d) 9.00% in the presence of LDHs in the cylindrical dialysis membrane and hydrogen peroxide in solution. The corresponding calibration curve, thus obtained, is shown in Figure 11. Lastly, the results of the analyses carried out, using disinfectant samples, are reported in Table 2.

Figure 11 also shows the representative points of the percentages of denatured ethanol found in disinfectant samples via linear interpolation on the calibration curve. The values, thus obtained, are compared with those declared by the manufacturer in Table 2. It can be observed how the percentage difference between the experimentally found values and those declared never exceeds approximately +5.7%. These percentage differences (all slightly positive) rise from the traces of other substances, as stated by the manufacturer, such as benzyl alcohol, contained in the tested samples, which are also oxidized inside the fuel cell. We can conclude that there is a more-than-acceptable agreement between the percentages of ethanol declared and those found within the limits of experimental precision, evaluated by carrying out at least three repeated measurements for each product and always yielding ≤4.5%.

## 4. Conclusions

In this research, we demonstrated how the presence of LDHs placed in the anodic section of a fuel cell can improve the performance of a simple direct catalytic ethanol fuel cell, since it helps to increase the speed of the oxidation reaction of the fuel (ethanol). The idea of using LDHs as a catalyst to increase the performance of a fuel cell, even if different from the one we used, i.e., (Zn^II^,Al^III^(OH)_2_)^+^NO_3_^−^·H_2_O, is not entirely new [41,42]. In other cases, certain types of LDHs have been used as an anion transport membrane inside a fuel cell [43,44]. The LDHs employed in these works are usually difficult to be synthesize, less pure, and often also much more toxic than ours. Furthermore, the methods of introducing LDHs into the anodic section of the fuel cell are much more complex than the one we employed, which uses a simple cylindrical dialysis membrane support. This technique, given its simplicity, is adaptable to most commercial fuel cells, without the need for complicated modifications. It also turned out to be quite efficient, as well as practice. It was also verified that a further increase in the sensitivity of the fuel cell modified with LDHs can be obtained, if one operates in the presence of H_2_O_2_. Lastly, we demonstrated that the use of a specific enzyme immobilized to LDHs through cross-linkage can significantly increase the oxidation efficiency of our fuel cell. Nevertheless, even if the use of an enzyme is not a new idea [16,45,46,47,48], we proved that the immobilization of the enzyme on the LDH and the insertion of this system in a small dialysis membrane is, indeed, an efficient and feasible possibility. In this way, we realized a practical system that makes a kind of breathalyzer for the determination of ethanol in human saliva. Finally, the simple modification of the anodic compartment of the fuel cell with LDHs, in the presence of hydrogen peroxide, made it possible to perform a sensitive analytical check for the determination of denatured ethanol content in commercial products, sold for thorough hand disinfection, i.e., for the purpose of protection against COVID-19.

Lastly, among the improvements achieved in this work from an analytical point of view, it should not be forgotten that the increase in the speed of the ethanol oxidation process in the fuel cell could also be extremely useful for the purposes of improving the energy performance of a DCEFC. In fact, we intend to dedicate a forthcoming more extensive study to this important problem and to carry out comparisons with very different fuel cells based, for example, on recent Solid Oxide Fuel Cell (SOFC) [49] systems, such as the Zn- Air Fuel Cell system [50] or methane-fueled SOFCs [51].

## Figures and Tables

**Table 1:** Comparison of the slope values of the tangent line at the point of maximum slope to the obtained curves, without and with LDHs (63 mg), at the different percentages of hydroalcoholic solution used.

% Ethanol	Slope without LDHs			Slope with LDHs		
	[V s^−1^]			[V s^−1^]		
1.25%	5.12 × 10^−5^	±	3.79 × 10^−6^	7.10 × 10^−5^	±	7.32 × 10^−6^
2.50%	1.76 × 10^−4^	±	2.10 × 10^−5^	3.17 × 10^−4^	±	3.83 × 10^−5^
5.00%	4.19 × 10^−4^	±	3.23 × 10^−5^	5.13 × 10^−4^	±	4.19 × 10^−5^
7.50%	8.35 × 10^−4^	±	1.65 × 10^−5^	1.15 × 10^−3^	±	5.95 × 10^−5^
10.0%	9.87 × 10^−4^	±	1.85 × 10^−5^	1.24 × 10^−3^	±	5.34 × 10^−5^
15.0%	1.34 × 10^−3^	±	6.37 × 10^−5^	1.39 × 10^−3^	±	9.55 × 10^−5^

**Table 2:** Results of ethanol determination in real samples: two human saliva (named BS1 and BS2) and three diluted (1:20) disinfectant solutions (named DS1, DS2, and DS3).

Samples	(a) % Nominal Final Values Added in the Sample	(b) % Experimental Found Value (RSD % ≤ 4.0)	∆% = [(b − a)/a]%
BS1	0.07	0.073	+4.3
BS2	0.17	0.167	−1.8
	**% Nominal Values Reported by Producer**	**% Experimental Found Value** **(RSD % ≤ 4.5)**	**∆% = [(b − a)/a]%**
DS1	3.30	3.49	5.76
DS2	3.50	3.70	5.71
DS3	3.70	3.87	4.59

BS = Biological Sample (human saliva), DS = Disinfectant Sample.

## Data Availability

Not applicable.
